# Identifying the determinants and associated factors of mortality under age five in Ethiopia

**DOI:** 10.1186/s12889-021-10157-5

**Published:** 2021-01-28

**Authors:** Hunachew Kibret Yohannis, Moges Zerihun Fetene, Habtamu Gebremariam Gebresilassie

**Affiliations:** 1 Statistics Department, Under Natural and Computational Science College, at the University of Gondar, Gondar, Ethiopia; 2 Statistics Department, Under Natural and Computational Science College, Dilla University, Dilla, Ethiopia

**Keywords:** Under-five mortality, Count regression models, Overdispersion, Ethiopia

## Abstract

**Background:**

Mortality rate under the age of five is the proportion of deaths of children below the age of 5 years out of 1000 live births. It is related with the living standard of a population, and it is taken as one of the health and socioeconomic status deterioration index. Mortality rate under the age of five also indicates a poor quality life standards of a population. It is very significantly high in Sub-Saharan African countries. Ethiopia is one of these Sub-Saharan African countries where mortality rate under the age five is high. This research work aims to identify the determinants and associated factors of under-five mortality in Ethiopia.

**Methods:**

The data for this paper were gathered from the EDHS 2016, collected by CSA. In this study, count family models such as Poisson, negative binomial, zero-inflated Poisson and zero-inflated negative binomial regression were applied for analyzing the data. Each of these count models were compared with different statistical tests like log-likelihood ratio test, Akaike information criteria, mean absolute difference, Vuong test and observed versus predicted probability plot.

**Results:**

The study revealed that as mothers’ age at first birth increased by one unit, the average number of under-five mortality rate decreased by 2.69%. In the same way the number of under-five mortality of Afar, Benishangul Gumuz and Dire Dawa were 1.3446, 1.6429 and 1.3320 times more likely to Tigray respectively. The risk of under-five mortality for primary and secondary education level of the mother was 28.31 and 40.96% less likely than to mothers who have no education respectively.

**Conclusion:**

From the result we found that, there were overabundance zeros and broad heterogeneity in the non-zero outcomes. Zero-inflated negative binomial regression model was found to best fit the data, and from the regression model, age of mothers at first birth, mother’s education level, place of residence and region were statistically significant factors of under-five mortality per mother.

## Background

Under-five mortality is related with the prosperity of a population and taken as one of the health and socioeconomic status improvement index and also indicates a quality of life of the population [1]. Decreasing under-five mortality is a global target and it is a Sustainable Development Goals (SDGs) key index. It is targeted to reduce under-five mortality rates below 25 deaths by 2030 [1]. Under-five mortality rate indicates proportion of children deaths occurring between birth and 5 years of age which is expressed out of 1000 live births [2].

Despite the global effort in reducing child mortality over the past few decades, an estimated 5.4 million children under age five died in 2017 [2]. Since 1990, considerable improvements have been made in child survival globally. However, improving child survival stays a matter of critical concern. Among 195 nations, 52 nations need quick progress of diminishing U5M. These nations are found in many parts of the world, and many of them are found in Sub-Saharan Africa. Among these nations 13 nations will not arrive at the objective until 2050 if existing under-five mortality rate trends go on. The greater number of these deaths are happening in sub-Saharan Africa. Besides Sub-Saharan African countries, around 30% are also happening in Southern Asia countries. The SDG goal is targeted to decrease the number of under-five mortality by 10 million between the years 2017 and 2030 [3].

There is a big difference in under-five mortality across the world nations. Sub-Saharan Africa countries take the greater number of under-five mortality rate on the planet. In 2016, the region had an average under-five mortality rate of 79 deaths for each 1000 live births. This means one child in thirteen deaths before his/her fifth years birthday celebration. It is fifteen times more than the mean proportion of one of every one hundred eighty nine in developed nations, or twenty times more than the proportion of 1 out of 250 in the region of Australia and New Zealand. The danger of death for a child conceived in the most noteworthy mortality nation is around 60 times higher than in the least mortality nation [4].

The Ethiopian Demographic and Health Survey showed 88 and 67 deaths for every 1000 live births in its 2011 and 2016 reports, respectively. The recent UNICEF report indicates that Ethiopia’s under-five mortality rate is 58 per 1000 live births. This figure implies that the under-five mortality rate in the base year 1990 was as high as 206 [1, 5]. Even if under-five mortality has declined in Ethiopia, there is substantial difference among regions of the country [6]. The different regions of the country have diversified lifestyle, culture, ethnic or environmental determinants. Because of the existence of potential cultural, socioeconomic and environmental differences among the population of the country (Tibebu, 2011).

In Ethiopia, the risk of under-five mortality varies by: household income category, level of mother’s education, inadequate access to health services, lack of safe drinking water and sanitation services, poor nutrition and place of residence (MoFED and UNDP, 2012).

Even though there is a research on Poisson and negative binomial regression models such as Tibebu (2011) on multilevel count model, still there is a gap on assessing excess zeros. Hence, under-five mortality is not occurred for all of the women. There is a probability of getting excess zeros, and this situation is solved by fitting zero-inflated Poisson and zero-inflated negative binomial regression models. Therefore, this study is focused on filing this research gap by fitting zero-inflated count statistical regression models of under-five mortality. At the end of the day it is expected to improve the understanding of many people who have the chance of reading the paper on under-five mortality situations in Ethiopia. It also serves as base line information to other researchers for further studies on under-five mortality. The main aim of the study is to identify the determinants and associated factors of under -five mortality in Ethiopia.

## Methods

### Data source

Ethiopian Demographic and Health Survey which was conducted in 2016 by Central Statistical Agency (CSA) was the data source for this paper. The survey was conducted for the fourth time. It covers the rural and urban parts of the nine regions of Ethiopia and the two administrative cities, Dire Dawa and Addis Ababa. The main aim of 2016 Ethiopian Demographic and Health Survey was to present up to date and trusted data on different demographic, environmental, economic, and health related issues [7]. This study analyzed event of 10,283 women aged 15–49 years on the number of under-five mortality that the mother has encountered. The data sets analyzed during the current study are publicly available in the Ethiopian Demographic and Health Survey (EDHS) available at DHS website (http://dhsprogram.com).

### Response variable of the study

The number of deaths of children aged from birth up to 59 months that each mother had experienced in her reproductive life time is the variable of interest or the dependent variable (Y_i_) for this research, and it assumes the values 0, 1, 2, 3, …

### Data analysis methods

In research work, it is desirable to align the type of data with an appropriate statistical model. In line with this, count regression models are not similar with normal linear regression models. Hence, count models possess non-negative discrete and nonlinear values [8]. Poisson regression model is considered as an initial statistical model in count families. So, it fits well with the attribute of count variable [9, 10].

Even though Poisson regression model has an advantage, it has also a potential drawback. Its drawback is an equidispersion of the same mean and variance assumption. When equidispersion assumption is not satisfied, the mean of actual counts is less than the variance, overdispersion takes place. The problem of not controlling overdispersion in the model is that it leads to exaggerated test statistic, unfair standard errors. It also makes the estimates become inconsistent. Usually, it is a common task to overcome the problem of overdispersion after fitting Poisson regression model using either Quasi Likelihood estimation method which is developed by Wedderburn [11] or negative binomial regression model (NBRM) [12, 13].

Negative binomial regression model is an immediate expansion of Poisson regression model, and it can solve overdispersion problem [14, 15]. Moreover, negative binomial regression model estimates the dispersion parameter, and it also specifies the mean and the variance independently [16]. However, in Poisson regression model, the dispersion parameter that connects the variance and the mean set at one. Here we understand that variance is the only dissimilarity between negative binomial and Poisson regression models while the model coefficients can be given to be synonyms across the two specified models. If there is a variability on the variance, it is obvious that the standard errors of the two models are also different. When there is an existence of overdispersion on the response variable, then the variance becomes larger which interne shows that the standard errors are also larger even if it is more proper [17, 18].

The inclusion of dispersion parameter in negative binomial regression model has an advantage of equipping overdispersion by controlling undetected variability in count data. The overdispersion problem arises due to hidden heterogeneity and availability of excess zeros in the data [19]. Even though overdispersion and excess zeros are problems, negative binomial regression model and zero inflated Poisson and zero inflated negative binomial regression models respectively considered as an immediate solutions. In most practical instances, count type of data exhibits the property of rightly skewed, non-negative, overdispersed and have maximum zeros. Hence, this study furnishes an applied formulation of fitting count data directing on data that possess overdispersion and excess zeroes.

### Zero-inflated Poisson and zero-inflated negative binomial regression models

Since there are so many zeros available from the collected data, we applied zero-inflated Poisson and zero-inflated negative binomial regression models to model the collected data [20]. Zero-inflated negative binomial regression model is one of the regression models under the family of count regression models and an extended part of negative binomial regression model [21].

The major difference between zero-inflated Poisson regression model and zero-inflated negative binomial regression model is that just like the replacement of negative binomial regression model by Poisson regression model. Which means a simple adjustment of zero-inflated Poisson regression model gives us zero-inflated negative binomial regression model [22]. The probability mass function is given by:

$$ \mathrm{p}\left({\mathrm{y}}_{\mathrm{i}}\right)=\left\{\begin{array}{l}{\Phi}_{\mathrm{i}}+\left(1-{\Phi}_{\mathrm{i}}\right){\left(1+{\upalpha \uplambda}_{\mathrm{i}}\right)}^{\raisebox{1ex}{$-1$}\!\left/ \!\raisebox{-1ex}{$\upalpha $}\right.}\kern0.72em \mathrm{if}\;{\mathrm{y}}_{\mathrm{i}}=0\\ {}\left(1-{\Phi}_{\mathrm{i}}\right)\frac{\Gamma \left({\mathrm{y}}_{\mathrm{i}}+\frac{1}{\upalpha}\right){\left({\upalpha \uplambda}_{\mathrm{i}}\right)}^{{\mathrm{y}}_{\mathrm{i}}}}{\Gamma \left({\mathrm{y}}_{\mathrm{i}}+1\right)\Gamma \left(\frac{1}{\upalpha}\right){\left(1+{\upalpha \uplambda}_{\mathrm{i}}\right)}^{{\mathrm{y}}_{\mathrm{i}}+\frac{1}{\upalpha}}}\end{array}\right.\kern1.08em \mathrm{if}\;{\mathrm{y}}_{\mathrm{i}}>0 $$

where λ_i_ is the mean value of the non-zero group and Φ_i_ is the probability of the zero groups of the outcome variable that can be modeled with the related independent variables [23].

The zero-inflated negative binomial regression model has the variance of, var. (y_i_) = λ_i_ (1-Φ_i_) (1 + αλ_i_ + Φ_i_λ_i_) and mean of E (y_i_) = λ_i_ (1-Φ_i_). The parameters like λ_i_ and Φ_i_ depend on explanatory variables and the value of alpha (α) is a scalar with the value of greater than or equals to zero. Hence, the existence of overdispersion satisfies when either the value of Φ_i_ or α is greater than zero. Thus, the above equation of zero-inflated negative binomial distribution reduced to negative binomial distribution when the parameter Φ_i_ equals zero and in the same way when the value of α becomes zero. Therefore, the above ZINB distribution reduced to zero-inflated Poisson distribution [20].

The likelihood function for estimating the zero-inflated negative binomial regression model parameters are given by [24].

$$ \mathrm{L}={\prod}_{{\mathrm{y}}_{\mathrm{i}}=0}\left\{{\Phi}_{\mathrm{i}}+\left(1-{\Phi}_{\mathrm{i}}\right){\left(1+{\upalpha \uplambda}_{\mathrm{i}}\right)}^{\frac{1}{\upalpha}}\right\}{\prod}_{{\mathrm{y}}_{\mathrm{i}}\ne 0}\left\{\left(1-{\Phi}_{\mathrm{i}}\right)\frac{\Gamma \left({\mathrm{y}}_{\mathrm{i}}+\frac{1}{\upalpha}\right){\left({\upalpha \uplambda}_{\mathrm{i}}\right)}^{{\mathrm{y}}_{\mathrm{i}}}}{\Gamma \left({\mathrm{y}}_{\mathrm{i}}+1\right)\Gamma \left(\frac{1}{\upalpha}\right){\left(1+{\upalpha \uplambda}_{\mathrm{i}}\right)}^{{\mathrm{y}}_{\mathrm{i}}+\frac{1}{\upalpha}}}\right\} $$

Taking the logarithm of both sides of the likelihood equation and after some adjustment are added, it is given by:

$$ \ln \left(L\left(\lambda, \alpha, \Phi, y\right)\right)={\sum}_i\left\{\begin{array}{l}I\left({y}_i\right)\ln \left({\varPhi}_i+\left(1-{\varPhi}_i\right){\left(1-{\alpha \lambda}_i\right)}^{\frac{-1}{\alpha }}\right)+\left[1-I\left({y}_i\right)\right]\Big(\ln \left(1-{\varPhi}_i\right)-{y}_i\ln \left(1+\frac{1}{{\alpha \lambda}_i}\right)-\frac{1}{\alpha}\ln \left(1+{\alpha \lambda}_i\right)\\ {}-\ln \varGamma \left({y}_i+\frac{1}{\alpha}\right)-\ln \varGamma \left(\frac{1}{\alpha}\right)-\ln \varGamma \Big(\left({y}_i+1\right)\end{array}\right\} $$

Finally, using EM algorithm we can estimate the unknown parameters of the model.

### Model comparisons for under-five mortality

The response variable in this research is the quantity of under-five mortality for every mother in her life time. Such sort of information (data) is modeled using count regression models. In this study, different possible count data models were considered. To distinguish the most proper and well fitted count regression model for the collected data, log-likelihood ratio test, Akaike information criteria, sum of mean absolute difference and Pearson values, Vuong test and observed versus predicted probability plot were used [8, 25–30].

In this study, to choose a best fitting model, four unique statistical models were considered. These are Poisson regression model, negative binomial regression model, zero-inflated Poisson and zero-inflated negative binomial regression models.

## Results

### Descriptive statistics

The data was analyzed on mothers of reproductive age in the study area. Out of the overall women considered in the sample, 29% of the women in the reproductive age have faced at least one under-five mortality in their lifetimes.

Table 1 indicates that the number and level of under-five mortality that the mothers in the sample have faced in their lifespan. Enormous quantities of under-five mortality for each mother were less often watched, which is strongly skewed to the right with excess zeroes. This is an indication of count data models with excess zeroes may be take into account.

**Table 1:** Number of mothers that were considered in under-five mortality

Number of under-five mortality per mother	Number of mothers		Total	Percentage
	Urban	Rural		
0	2275	5030	7305	71.04
1	317	1484	1801	17.52
2	91	624	715	6.95
3	29	247	276	2.68
4	10	104	114	1.11
5	7	32	39	0.38
6	2	18	20	0.19
7	1	5	6	0.06
> = 8	0	7	7	0.07
**Total**	**2732**	**7551**	**10,283**	**100**

**Fig. 1:**
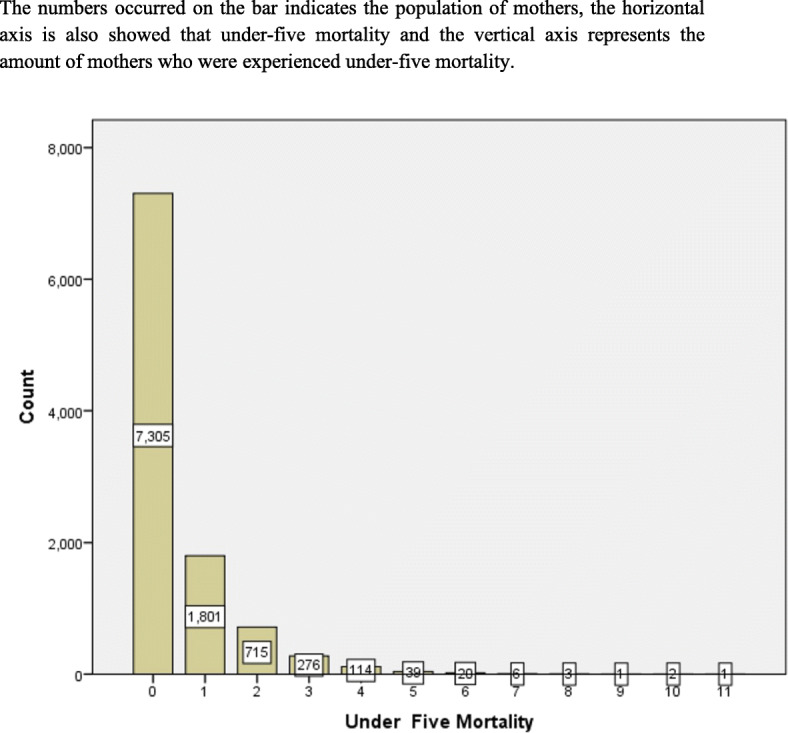
A bar chart of the quantity of Under-five mortality for every mother. On here the y-axis presented observed versus predicted probabilities and the x-axis labeled as the number of under-five mortality with four different colors and symbols of the four candidate count regression models

The simple bar chart below displayed as Fig. 1 showed the distribution of the quantity of under-five mortality for each mother. Since the number of zero results are excessively observed, the bar diagram is exceptionally crested at the absolute starting point and it steps down to the right. Generally, it is skewed to the right bar chart. In any case, huge quantities of under-five mortality for every mother were less habitually watched. This perhaps is an indication that count data models with excess zeroes may be take on into account.

Table 2 displayed the general overview of the explanatory variables that directly influence the risk of under-five mortality. The variables which are included in the study are place of residence, region, age at first birth, mass media follow up, education of mother, source of drinking water, availability of toilet facility, wealth index and mother’s religion.

**Table 2:** Synopsis insights of some significant factors identified with under-five mortality

Variables with category		Mean	St. Deviation	Percentages of U5M > =1	Median
Place of residence	Urban	0.25	0.671	16.73%	0
	Rural	0.56	1.027	33.39%	0
Region	Tigray	0.40	0.838	25.54%	0
	Afar	0.66	1.101	36.20%	0
	Amhara	0.54	0.972	32.87%	0
	Oromia	0.47	0.925	29.84%	0
	Somali	0.61	1.069	34.80%	0
	Benishangul	0.61	1.189	31.72%	0
	SNNPR	0.56	0.991	34.07%	0
	Gambela	0.39	0.742	26.26%	0
	Harari	0.32	0.711	21.78%	0
	Addis Ababa	0.12	0.403	9.90%	0
	Dire Dawa	0.45	0.956	26.65%	0
Mass media follow up	No	0.56	1.016	32.92%	0
	Yes	0.27	0.688	18.74%	0
Education level of mothers	No education	0.65	1.193	37.03%	0
	Primary	0.27	0.631	19.77%	0
	Secondary and above	0.13	0.412	10.51%	0
Drinking water source	Other wise	0.57	1.022	33.40%	0
	Piped water	0.34	0.778	21.48%	0
Availability of toilet facility	No toilet facility	0.57	1.011	33.38%	0
	Have toilet facility	0.42	0.894	26.03%	0
Wealth index	Poor	0.60	1.048	35.05%	0
	Middle	0.52	0.993	31.40%	0
	Rich	0.33	0.779	21.67%	0
Mothers religion	Orthodox	0.38	0.811	24.36%	0
	Protestant	0.44	0.898	27.36%	0
	Muslim	0.58	1.057	33.50%	0
	Others	0.53	0.934	32.47%	0

**Note:** St.Deviation: Standard Deviation

The overall number of mothers considered are 10,283 of which 2978 of them encountered under-five mortality. Generally, 16.73% of urban women faced with the problem of under-five mortality, and it was very small when compared with rural women residents which is 33.39%. The regional variation of under-five mortality per mother occurred the largest in Afar regional state (36.20%) compared with the smallest event in Addis Ababa administrative city (9.90%). All the others are in between the two. Regarding to mass media follow up, the event of under-five mortality was happened to be 18.74% for mothers who follow mass media but for mothers who didn’t develop the habit of following mass media scored 32.92% under-five mortality.

From a theoretical perspective, education of mother is an important determinant factor of under-five survival. Different literatures support this idea. Accordingly, mothers with secondary and above educational level have lowest under-five mortality (10.51%), but mothers who have no educational status have the maximum under five mortality (37.03%).

When we assess drinking water source and sanitation, piped water supply reduces under-five mortality. It reduces especially infant mortality directly by reducing the incidence of diarrhea that arises from the ingestion of contaminated water and food, and indirectly when care givers are able to devote more time to childcare instead of water collection activities. So that, households with piped drinking water source accounted below one over fourth under-five mortality per mother (21.48%) where as households with no piped water source scored 33.40% amount of the event. Large figure of under-five mortality was happened on households without any toilet (33.38%) as compared to households with toilet facility (26.03%).

Previous studies show that wealthier families can provide better nutrition, shelter and health services to children, which lead to an increase in child survival. According to this study among the three wealth index, poor families have largest under-five mortality per mother with the value of 35.05% as compared to rich and middle wealth families.

### Goodness of fit of the model and test of Overdispersion

Decency of attack of the fitted Poisson regression model was evaluated using Pearson based Chi-square test. The Pearson goodness of fit test of 10,530.7 at 10260 degree of freedom with p-estimation of (*p* = 0.0302) which would infer solid match for the data. In the event that the Pearson Chi-square worth partitioned by its degree of freedom is somewhat more noteworthy than one, that indicates over dispersion in the data. This result on Table 3, showed that there is a sign of overdispersion. Moreover, it is desirable to apply a formal statistical test of overdispersion. The value of the likelihood-ratio test of dispersion parameter alpha was Chi-square = 233.76 with p-estimation of < 0.0001 which demonstrated that there is an overdispersion in our data. Moreover, we can use lan of alpha estimation of the proportion test at one which is the chi-square value at one degree of freedom 463.14 with p-estimation of < 0.0001 it is significant. Along these lines, the observed data were better clarified by the negative binomial regression model than the Poisson regression model. In accordance with this, the negative binomial regression model was an amended fit than the Poisson regression model as one can see from Table 3, since Akaike information criteria (AIC) (16,121.2) and Bayesian information criterion (BIC) (16,294.92) values are small.

**Table 3:** Examination of Poisson and negative binomial regression models

Test	Estimate	Poisson	Negative Binomial
Pearson chi-square	Value	10,530.7	
	Degree of freedom	10,260	
	Value/df	1.03	
Akaike Information Criteria (AIC)		16,352.96	16,121.2
Bayesian Information Criterion (BIC)		16,519.44	16,294.92
Log likelihood		− 8153.4791	− 8036.6005
Likelihood ratio (LR) test	Value = 233.76 df = 1 *p*-value < 0.0001		

So far existence of overdispersion was assessed. Now it is time to check the cause of overdispersion. It might have happened due to variability of data or extra of zeros. In case of overdispersion, zero-inflated Poisson regression model typically modeled finer than a regular Poisson regression model. At the point when the significant wellspring of overdispersion is a dominance of zero tallies, the subsequent overdispersion cannot be modeled precisely with the negative binomial regression model. An elective path for demonstrating this kind of data is the zero-inflated Poisson or zero-inflated negative binomial regression model which considers the excess of zeroes.

### Model selection

As shown in the summery Table 4, we can compare all fitted models based on maximum difference, mean absolute difference, log-likelihood and AIC values. The model with the smallest mean absolute difference and AIC value, at the same time the model with largest maximum difference and likelihood ratio is preferred. When we see the absolute mean difference, ZINB model is preferred, because it has smallest mean absolute difference value. In other words, by considering AIC value, ZINB is also preferable. Since ZINB has largest log-likelihood value and maximum difference, in line with this ZINB model is the most appropriate and preferable model among the four models.

**Table 4:** Fit statistic of count regression models

Count regression models				
Criteria	PRM	NBRM	ZIP	ZINB
Maximum Difference	−0.095	−0.008	−0.014	−0.006
At value	1	1	2	1
Mean |Diff|	0.020	0.002	0.004	0.001
Log-Likelihood	− 9700.316	− 9098.637	− 9147.549	− 9071.791
AIC	19,444.632	18,243.275	18,383.098	18,233.582

For non-nested models as shown on Table 5: ZIP versus Poisson and ZINB versus NB regression models were identified using the Vuong test statistic. At the same time, for nested models which is displayed on Table 6, Poisson versus NB and ZIP versus ZINB regression models could be identified by using likelihoods ratio test.

**Table 5:** Model comparisons using Vuong test for non-nested models

Models	Vuong Statistics(V)	***p***-values	Preferred model
ZIP Vs Poisson	13.768	< 0.001	ZIP
ZINB Vs NB	3.758	< 0.001	ZINB

**Table 6:** Model comparisons using Likelihood ratio test for nested models

Models	likelihood ratio test	p-value	preferred model
Poisson vs NB	1203.357	< 0.001	NB
ZIP Vs ZINB	151.516	< 0.001	ZINB

Table 7 indicates that we have actual or observed values, predicted probability, absolute difference between actual and predicted probability values and Pearson values of the four count models. When we observe the sum of mean absolute difference, ZINB model had the minimum values from the other three models. In addition to this; among the four models, the one which has the smallest sum of Pearson value is the best model. From the table, ZINB has the minimum Pearson sum of the predicted and actual probabilities than other count models. However, negative binomial regression model seems to have the smallest Pearson sum and mean absolute difference but it doesn’t perform as much as ZINB regression model done. This might be an indication of there is improvement in ZINB regression model than negative binomial regression model.

**Table 7:** Model comparison by sum of predicted and actual probabilities

Count	Actual	Predicted probability				|difference|				Pearson			
		PRM	NBRM	ZIP	ZINB	PRM	NBRM	ZIP	ZINB	PRM	NBRM	ZIP	ZINB
0	0.710	0.636	0.708	0.710	0.709	0.074	0.002	0.000	0.002	88.714	0.063	0.000	0.044
1	0.175	0.270	0.183	0.165	0.181	0.095	0.008	0.014	0.006	341.521	3.731	11.226	2.198
2	0.070	0.075	0.064	0.083	0.065	0.005	0.006	0.014	0.004	4.124	5.067	23.313	2.655
3	0.027	0.016	0.025	0.032	0.026	0.011	0.002	0.005	0.001	79.459	1.044	8.114	0.498
4	0.011	0.003	0.011	0.010	0.011	0.008	0.000	0.001	0.000	274.792	0.188	1.772	0.193
5	0.004	0.000	0.005	0.003	0.005	0.003	0.001	0.001	0.001	323.641	1.692	6.573	1.294
6	0.002	0.000	0.002	0.001	0.002	0.002	0.000	0.001	0.000	834.041	0.120	34.978	0.012
7	0.001	0.000	0.001	0.000	0.001	0.001	0.000	0.000	0.000	737.504	1.538	20.738	1.082
8	0.000	0.000	0.000	0.000	0.000	0.000	0.000	0.000	0.000	1997.168	0.557	38.723	0.317
9	0.000	0.000	0.000	0.000	0.000	0.000	0.000	0.000	0.000	2630.227	0.623	28.573	0.431
**sum**	**1.000**	**1.000**	**1.000**	**1.000**	**1.000**	**0.200**	**0.020**	**0.037**	**0.015**	**7311.192**	**14.622**	**174.510**	**8.724**

**Fig. 2:**
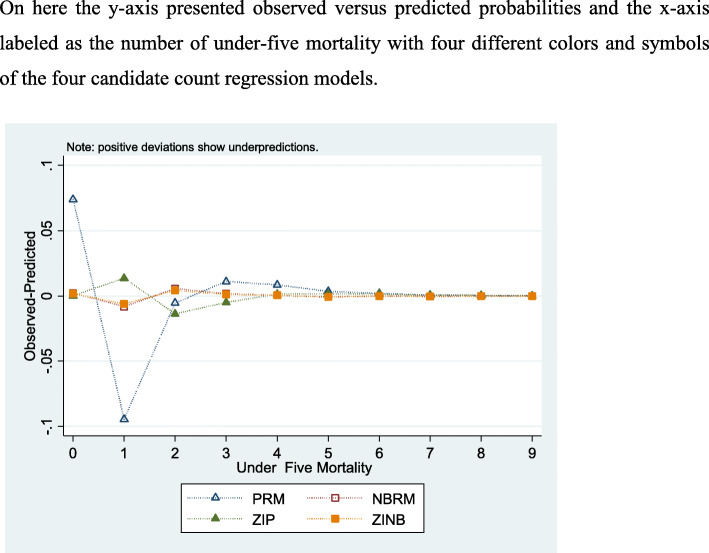
Residual plots for estimated models

In addition, the model would be compared by using observed versus predicted probability plot. Hence, the residual plot in Fig. 2 confirms that ZINB model fits well the data and it is the most appropriate model among the four count models because almost all ZINB points pass through 0 and makes a straight line after some moments of under-five mortality and if we compare the models on the graph ZINB regression model best approaches the zero line.

## Discussion

The finding of the study for non-zero group at Table 8 showed that, age of mothers at first birth was found to be a significant factor on under-five mortality. By keeping other variables held constant in the model, a one unit increase of age at first birth will decrease the average number of under-five mortality by 2.69% in Ethiopia (IRR = 0.9731; CI = 0.9638, 0.9825). With regard to region, the number of under-five mortality per mother has risk for those mothers who live in Afar, Benishangul Gumuz regional state and Dire Dawa city administration. The risk of under-five mortality for those mothers who live in Afar regional state was 1.3446 (IRR = 1.3446; CI = 1.0514, 1.7196) times more likely to die before age five as compared to those mothers who lives in Tigray regional state keeping other variables held constant in the model. Similarly, by keeping other variables held constant in the model, the risk of under-five mortality for those mothers who live in Benishangul Gumuz regional state was 1.6429 (IRR = 1.6429; CI = 1.2891, 2.0938) times more likely to die before age five as compared to those mothers who live in Tigray regional state. In the same way, mothers who live in Dire Dawa city administration was 1.3320 (IRR = 1.3320; CI = 1.0136, 1.7505) times more likely to die before age five as compared to those mothers who live in Tigray regional state keeping other variables held constant in the model. According to the result of this study, mother’s education level was found to be statistically significant factor for under-five mortality in Ethiopia. The result indicates that the risk of under-five mortality was 28.31% (IRR = 0.7169; CI = 0.6552, 0.7844) less likely for mothers who has primary education level as compared to mothers who have no education level keeping other variables held constant in the model. Similarly, the risk of under- five mortality was 40.96% (IRR = 0.5904; CI = 0.4940, 0.7056) less likely for mothers who has secondary and above education level as compared to mothers who have no education level keeping other variable held constant in the model.

**Table 8:** ZINB regression model parameter estimation for none zero group

Explanatory variables	Coef.	Z	P > |Z|	IRR	95% CI for IRR	
					Lower	Upper
Age	−0.0273	−5.58	0.000	0.9731	0.9638	0.9825
Place (Urban)						
Rural	−0.1265	−1.50	0.133	0.8812	0.7469	1.0395
Region (Tigray)						
Afar	0.2661	2.36	0.018	1.3446	1.0514	1.7196
Amhara	0.1739	1.66	0.098	1.1900	0.9686	1.4618
Oromia	0.0409	0.35	0.727	1.0417	0.8279	1.3108
Somali	0.1463	1.16	0.247	1.1575	0.9035	1.4829
Benshangul	0.4965	4.01	0.000	1.6429	1.2891	2.0938
SNNPR	0.2187	1.94	0.053	1.2444	0.9976	1.5524
Gambela	0.1218	0.83	0.404	1.1295	0.8485	1.5035
Harari	0.0818	0.51	0.612	1.0852	0.7913	1.4884
Addis Ababa	−0.0432	− 0.15	0.882	0.9577	0.5408	1.6960
Dire Dawa	0.2867	2.06	0.040	1.3320	1.0136	1.7505
Mass Media (No)						
Yes	−0.0113	− 0.22	0.825	0.9888	0.8948	1.0926
Education level (No education)						
Primary	−0.3328	−7.25	0.000	0.7169	0.6552	0.7844
Secondary and above	−0.5269	−5.79	0.000	0.5904	0.4940	0.7056
Source of drinking water (others)						
Piped water	−0.0118	− 0.27	0.786	0.9883	0.9079	1.0758
Toilet facility (No)						
Yes	−0.0250	−0.61	0.541	0.9753	0.9003	1.0566
Wealth index (Poor)						
Middle	−0.0368	−0.73	0.466	0.9638	0.8732	1.0640
Rich	−0.0758	−1.52	0.130	0.9270	0.8405	1.0225
Religion (Orthodox)						
Protestant	0.0330	0.38	0.701	1.0336	0.8731	1.2235
Muslim	0.0559	0.75	0.456	1.0575	0.9131	1.2246
Others	0.0140	0.08	0.933	1.0141	0.7327	1.4034
**Constant**	**−1.3697**	**−9.47**	**0.000**	**0.2542**	**0.1915**	**0.3375**
Lantotal	1 (offset)			1		

Note: *IRR* Incidence Rate Ratio; *OR* Odds Ratio; *CI* Confidence Interval; variable categories on the parenthesis are reference group; *Coef.* Coefficients (β); *Z* Z-calculated value; *P* P-value

The result of this study for inflated group as displayed on Table 9, showed that place of residence was found to be statistically significant factor for under-five mortality in Ethiopia. By keeping other variables held constant in the model, the risk of under-five mortality was 44.03% (OR = 0.5597; CI = 0.3379, 0.9272) times less than for mothers who live in rural part of Ethiopia as compared to mothers who live in urban part of Ethiopia. The finding of this study showed that the number of under-five mortality per mother has risk for those mothers who live in Benishangul Gumuz regional state. By keeping other variables held constant in the model, the risk of under-five mortality those mothers live in Benishangul Gumuz regional state was 2.7008 (OR = 2.7008; CI = 1.1008, 6.6261) times greater than those mothers who lives in Tigray regional state.

**Table 9:** ZINB regression model parameter estimation for inflate group

Explanatory varaibles	Coef.	Z	P > |Z|	OR	95% CI for OR	
					Lower	Upper
**Place (Urban)**						
Rural	−0.5803	−2.25	0.024	0.5597	0.3379	0.9272
**Region (Tigray)**						
Afar	0.3457	0.66	0.508	0.3457	−0.6767	1.3682
Amhara	−0.3994	−0.74	0.461	−0.3994	−1.4615	0.6627
Oromia	0.1257	0.26	0.798	0.1257	−0.8368	1.0881
Somali	0.6155	1.20	0.229	0.6155	−0.3876	1.6187
Benishangul	0.9935	2.17	0.030	2.7008	1.1008	6.6261
SNNPR	−0.2853	− 060	0.545	−0.2853	−1.2099	0.6393
Gambela	−0.5331	−0.78	0.437	−0.5331	−1.8767	0.8106
Harari	0.6205	1.07	0.284	0.6205	−0.5148	1.7559
Addis Ababa	0.4290	0.57	0.571	0.4290	−1.0565	1.9144
Dire Dawa	0.2480	0.45	0.650	0.2480	−0.8224	1.3182
**Religion (Orthodox)**						
Protestant	0.3565	1.24	0.214	0.3565	−0.2054	0.9184
Muslim	−0.3654	−1.28	0.199	−0.3654	−0.9231	0.1922
Others	−0.1457	−0.22	0.827	−0.1457	−1.4535	1.1620
**Cons**	−0.8277	−2.07	0.039	0.4371	0.1993	0.9585
**Lnalpha**	−2.5180	−5.82	0.000	−2.5180	−3.3665	−1.6694
**Alpha**	0.0806			0.0806	0.0345	0.1884

Note: *IRR* Incidence Rate Ratio; *OR* Odds Ratio; *CI* Confidence Interval; variable categories on the parenthesis are reference group; *Coef.* Coefficients (β); *Z* Z-calculated value; *P* P-value

## Conclusions

This study was designed to identify the most important under-five mortality variables through count regression models in Ethiopia. The study also identifies the best count fit model in order to analyze under-five mortality data. The data were taken from [7] Ethiopian Demographic and Health Survey data. In this study, 10,283 women were taken, out of which 71% of the mothers have not faced any under-five mortality in their lifetime. This implies that using zero inflated model is appropriate to fit this data set. From the exploratory results we could identify that there is an excess zeros and high variability in the non-zero values. The variance of the number of under-five mortality was larger than its mean, indicating that there was possibility of overdispersion.

The appropriate fitted model was selected from among different candidate models like Poisson, negative binomial (NB), zero-inflated Poisson (ZIP) and zero-inflated negative binomial (ZINB) regression models using different comparison techniques. The comparison was conducted through log-likelihood ratio test (LRT), Akaike information criteria (AIC), mean absolute difference, Vuong test and observed versus predicted probability plot. LRT which is used to compare any two nested models such as Poisson versus negative binomial (NB) and zero-inflated Poisson (ZIP) versus zero-inflated negative binomial (ZINB) was used. Non-nested models such as Poisson versus ZIP and NB versus ZINB were compared using Vuong test.

Since the under-five mortality data in Ethiopia contains an excess zero, the standard Poisson and negative binomial regression models were not enough to fit the data well. Zero-inflated negative binomial model was appropriate to fit the number of under-five mortality data due to the presence of excess zero in the data. In addition to this, the violation of Poisson model assumption that is the mean is smaller than the variance of number of under-five mortality.

This study also involves predictor variables that had significant effects on number of under-five mortality per mother in Ethiopia. For selected ZINB model, for the non-zero group, the predictor variables like age of mothers at first birth, region, mother’s education level were statistically significant factors while for the zero group, place of residence and region were statistically significant factor on the number of under-five mortality per mother in Ethiopia.

### Limitation of the study

Some variables were not included in the study due to the presence of high missing values such as: number of antenatal visits for pregnancy, preceding birth interval, smoking cigarettes, duration of breastfeeding, chewing chat etc. Since this study was based on secondary data from EDHS 2016, we try to study only the variables which are included in the questionnaire. In addition, the study used reported characteristics of mothers and households that may vary with time. Mothers’ age at first birth was fixed but others are time-varying covariates. However, in this analysis, all covariates were considered as fixed during the study period. Moreover, only surviving women age 15–49 were interviewed. Therefore, no data were available for children of women who had died.

## Data Availability

All the data are publicly accessible on Ethiopian Demographic and Health Survey (EDHS) available at DHS website (http://dhsprogram.com).

## Abbreviations

AIC: Akaike Information Criteria
BIC: Bayesian Information Criterion
EDHS: Ethiopian Demographic and Health Survey
HIV: Human Immune Virus
AIDS: Accrued Immune Deficiency syndrome
LRT: Likelihood Ratio Test
NBRM: Negative Binomial Regression Model
PRM: Poison Regression Model
SDGs: Sustainable Development Goals
U5M: Under-Five Mortality
ZINB: Zero-inflated Negative Binomial Regression
ZIP: Zero-Inflated Poisson
CSA: Central Statistical Agency
